# Evaluation of Abdominal Aortic diameter at different vertebral levels using Abdominal Computed Tomography Angiography in Nepalese adults: A retrospective cross-sectional study

**DOI:** 10.1097/MS9.0000000000003858

**Published:** 2025-09-12

**Authors:** Pradip Adhikari, Sumarg Simkhada, Roshan Chaudhary, Suraj Keshari, Archana Pandey, Madhav Sapkota

**Affiliations:** a Kathmandu University School of Medical Sciences, Dhulikhel Hospital, Dhulikhel, Nepal; b Kathmandu University School of Medical Sciences, Kathmandu, Nepal; c Kathmandu University School of Medical Sciences, Dhulikhel, Nepal; d Institute of Engineering, Tribhuwan University, Kathmandu, Nepal

**Keywords:** abdominal angiography, abdominal aortic aneurysm, abdominal aortic diameter, aortic reference values, Nepalese population, vascular imaging

## Abstract

**Background:**

Accurate diagnosis and management of abdominal aortic aneurysms (AAAs) require knowledge of the normal range of abdominal aortic diameter. Previous studies have shown geographical variations in aortic diameters, with smaller measurements in Asian populations and larger measurements in Oceania. However, specific reference values for the Nepalese population are lacking. Establishing these reference ranges is crucial for diagnosing aortic diseases and selecting appropriate graft sizes in vascular procedures.

**Methods:**

This retrospective study includes patients over 18 years of age who underwent contrast-enhanced computed tomography of the abdomen and pelvis between September 2023 and December 2023. A total of 225 patients (124 males, 101 females; mean age: 50.95 ± 17.62 years) were included. A radiotechnician reviewed the examinations under the guidance of two radiologists and one senior radiotechnician. The diameter of abdominal aorta (AA) at the 12th thoracic (T12) vertebral level and the 3rd lumbar (L3) vertebrae was measured for each examination, which was analyzed and correlated with different study variables such as age, gender, alcohol consumption, and diabetic status.

**Result:**

The study included 225 patients (124 males, 101 females; mean age, 50.95 ± 17.62 years (range 18–88 years). The mean diameter of the suprarenal AA measured at the T12 vertebral level was 2.06 cm (±0.28) in men and 1.93 cm (±0.26) in women. The mean diameter of the infrarenal aorta measured at the L3 vertebral level was 1.54 cm (±0.21) in men and 1.41 cm (±0.20) in women. The age and gender of participants were found to have a statistically significant association with the diameter of the suprarenal AA. Additionally, age, gender, alcohol consumption, and diabetic status of participants were found to be statistically significantly associated with diameter of the infrarenal AA.

**Conclusion:**

We established reference values for AA diameters in the Nepalese population. The mean aortic diameter of the Nepalese population seems to be smaller than the Western population but comparable to the South Asian population.

## Introduction

The abdominal aorta (AA), beginning at the hiatus of the diaphragm at the level of the twelfth thoracic vertebra (T12) and descending in the midline ending at the fourth lumbar vertebra (L4), where it bifurcates into the common iliac arteries, is the largest vessel in the abdominal cavity[1]. The 13-cm-long AA supplies the kidney, spleen, liver, and other tissues located in the abdominal cavity. The AA typically has a diameter of 1.5–2 cm, and a diameter above 3 cm is regarded as an abdominal aneurysmal disease[2]. The evaluation of diameter of the AA is necessary for the diagnosis of an abdominal aortic aneurysm (AAA), which is diagnosed when it is more than 50% larger than the normal diameter[1].

**HIGHLIGHTS**

- Although studies suggest that abdominal aorta diameter varies geographically, establishing its normal range is essential for the diagnosis of abdominal aortic aneurysm and selecting appropriate aortic graft sizes.
- This retrospective cross-sectional study analyzed abdominal aortic diameters using computed tomography angiography in 225 Nepalese adults.
- The mean suprarenal aortic diameter at T12 was 2.06 cm (±0.28) in men and 1.93 cm (±0.26) in women, while the infrarenal aortic diameter at L3 measured 1.54 cm (±0.21) in men and 1.41 cm (±0.20) in women.

AAA is often asymptomatic and may remain undetected. The mortality rate from AAA rupture can reach 88% if the condition goes undetected and continues to enlarge[3]. Since AAA has a high death rate when properly assessed and diagnosed, early detection and treatment are essential to prevent rupture[4]. The assessment of the normal abdominal aortic diameter range in Nepalese individuals can lead to a noteworthy reduction in mortality through timely identification and treatment. Additionally, the data obtained from this study may assist operating surgeons in preoperatively arranging/selecting prosthetic grafts for aortic disorders in this group. The present study aims to establish reference values for suprarenal and infrarenal abdominal aortic diameters in the adult Nepalese population, measured at the T12 and L3 vertebral levels, respectively. It also seeks to examine the association between aortic diameter and demographic and health-related factors, including age, sex, alcohol consumption, and diabetic status.

This cross-sectional study has been reported in line with the STROCSS 2025 Guideline[5].

## Methods

### Study setting and patient selection

This is a descriptive, cross-sectional study conducted among patients at the Department of Radiology of a tertiary care hospital in Nepal. Data from September 2023 to December 2023 were collected after approval from the Institutional Review Committee. Written consent was obtained from the study participants for the collection and publication of their information and images.

During the study period of 4 months, 225 patients (124 males, 101 females; mean age, 50.95 ± 17.62 (range 18–88 years)) were enrolled in the study who were referred to the Department of Radiology for a contrast-enhanced computed tomography (CECT) of the abdomen and pelvis from various departments of the hospital. This study included all Nepali citizens aged 18 years and above who underwent CECT of the abdomen and pelvis between September and December 2023. Patients with known factors that could alter normal aortic anatomy were excluded, including those with congenital aortic anomalies, a history of aortic surgery, arteriovenous malformations, Marfan syndrome, Ehlers–Danlos syndrome, vasculitis, significant cardiac disease, large abdominal masses distorting the aorta, and pregnancy. The primary outcome variable was the abdominal aortic diameter, measured in centimeters at two anatomical levels: the suprarenal region (T12 vertebral level) and the infrarenal region (L3 vertebral level). Independent variables included age (in years), sex (male/female), diabetic status (yes/no), and alcohol consumption status (yes/no), which were analyzed to determine their association with aortic diameter at both measurement sites.

### Image acquisition

CECT of the abdomen and pelvis was performed using a single 128 multi-slice SIEMENS SOMATOM-perspective CT system. Patients were requested to fast for at least 6 hours, and 500 ml of water was given orally as a neutral contrast, 20–60 minutes before the scan. No oral contrast media was administered. Scanning was then performed in the craniocaudal/caudocranial direction. Un-enhanced CT of the abdomen was performed first from the T12 vertebra through mid-pelvis, using 5 mm section thickness and a table speed of 15 mm per rotation. Subsequently, participants were given 70–80 ml of intravenous contrast media injection, each 1 ml consisting of 0.769 g of Iopromide United States Pharmacopeia (USP) (equivalent to 370 mg iodine). CECT was initiated 25–30 seconds after the injection to coincide with the arterial phase. Scans were obtained in the helical axial plane from the level of the dome of the diaphragm to the level of the symphysis pubis. Images were acquired and reconstructed in different planes, saved, and studied. After the image acquisition, image reconstruction was done through a medium smooth filter.

### Image interpretation

**Figure 1.**
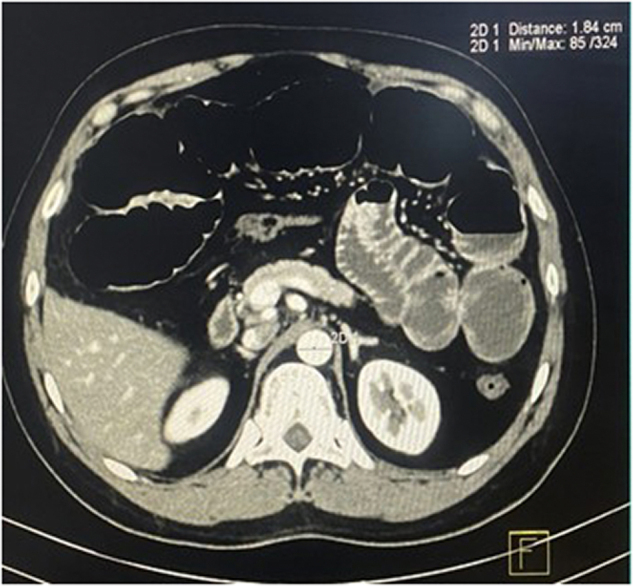
Diameter of abdominal aorta measured at T12 vertebral level.

**Figure 2.**
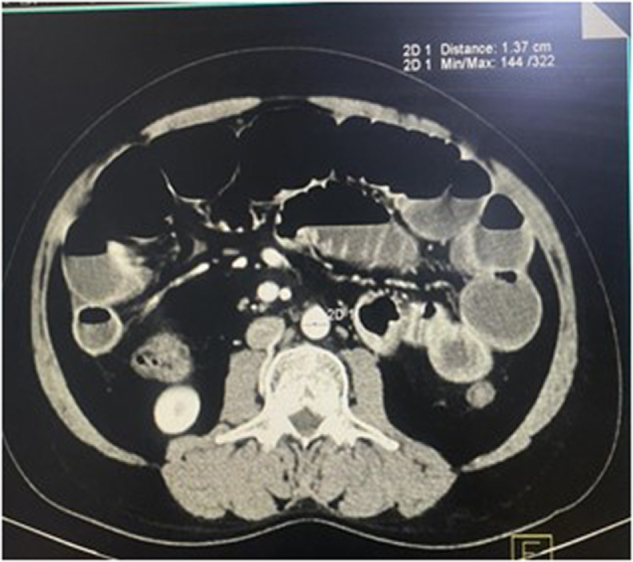
Diameter of abdominal aorta measured at L3 vertebral level.

The raw imaging data obtained were processed on a commercially available workstation for multiplanar reformations as well as 3D reconstruction with maximum intensity projection and volume rendering. All the measurements were performed by one radiotechnician under the supervision of two radiologists and one senior radiotechnician. Using the axial section at the level of the T12 vertebra (Fig. 1) and the L3 vertebra (Fig. 2), the reviewers measured the transverse diameter (TD) of the AA at both levels.

### Statistical analysis

The collected data were entered into MS Excel 2007 and analyzed using STATA version 15 (STATA Corporation, College Station, Texas 77845, USA) for statistical analysis. Descriptive statistics were calculated and presented as frequencies and percentages for categorical variables, while means and standard deviations were calculated for continuous variables. Using the multivariable linear regression models, the age of participants, gender, diabetic status of participants, and alcohol consumption of participants were adjusted to identify the association with the diameter of the AA at the T12 and L3 vertebral levels. Pearson’s correlation was used to test the correlations between variables.

## Results

### Demographic characteristics

Our study included a total of 225 participants. The mean age of the participants was 50.95 ± 17.62 years. Fifty-five percent of the participants were men, and 44.89% were female. Similarly, among the total respondent, the mean age was 50.95, with a minimum age of 18 and a maximum age was 88. Similarly, participants’ mean height was 1.56 m and mean body mass index (BMI) was 24.21, with a median value of 24.19 and a standard deviation of ±3.79. The minimum and maximum BMIs were 14.69 and 35.86, respectively. Among the respondent, 59 (26.2%) were smokers and 166 (73.8%) were non-smokers, and 66 (29.3%) of the participants were alcoholic and 159 (73.9%) were non-alcoholic. A total of 34 (15.11%) of the respondents had diabetes, and 191 (84.89%) were non-diabetic. Additionally, 29 (12.9%) were hypertensive patients and 196 (87.11%) were non-hypertensive patients. Participants’ demographics are summarized in Table 1. The mean diameter of the AA at the T12 vertebral level (suprarenal level) was 2.006 ± 0.28 cm, while at the L3 vertebral level (infrarenal level), it was 1.486 ± 0.22 cm (Table 2).

**Table 1:** Demographics of participants

Characteristics	Number of participants (*N* = 225)	Percentage (%)
Age (years)		
Mean ± SD (minimum–maximum)	50.95 ± 17.62 (18–88)	
Lower than 30	33	14.7%
30–49	66	29.3%
50–69	87	38.7%
70 or more	39	17.3%
Gender		
Male/female	124/101	55.1%/44.9%
Height		
Mean ± SD (minimum–maximum)	1.56 ± 0.11 (1.29–1.82)	
Less than 1.524	52	23.1%
1.524–1.6764	137	60.9%
1.6764 or more	36	16%
BMI		
Mean ± SD (minimum–maximum)	24.21 ± 3.79 (14.7–35.7)	
BMI less than 18.5	12	5.3%
18.5–26	140	62.2%
26–30	58	25.8%
30 or more	15	6.7%
Smokers (Yes/No)	59/166	26.2%/73.8%
Alcohol consumption (Yes/No)	66/159	29.3%/70.7%
Diabetic status of patients (Yes/No)	34/191	15.1%/84.9%
Hypertensive status of patients (Yes/No)	29/196	12.9%/87.1%

**Table 2:** Abdominal aorta diameters at T12 and L3 levels

Abdominal aorta level	Mean (±SD)	Median (min: max)	95% CI
T12	2.006 (±0.28)	2.01 (1.23–2.81)	1.96–2.04
L3	1.486 (±0.22)	1.48 (1.02–2.10)	1.45–1.51

### Clinical characteristics

The mean diameters of the AA at the suprarenal and infrarenal levels by patient characteristics are given in Tables 3 and 4, respectively. At the suprarenal level, both age and gender were significantly associated with abdominal aortic diameter (*P* < 0.05), with males and older individuals showing larger diameters. At the infrarenal level, age, gender, alcohol consumption, and diabetes status were all statistically significantly associated with aortic diameter (*P* < 0.05). For instance, participants with diabetes had smaller aortic diameters on average compared to non-diabetic individuals.

**Table 3:** **Differences in mean abdominal aorta diameters based on age and gender at** T12 **vertebral level**

Characteristics	Number	Mean (SD)	Mean difference (crude) (coef.)	Mean difference (adjusted) (coef.)	95% CI	*P*-value
Age of participants (years)						
Less than 30	33	1.68 (±0.21)	Ref	Ref	Ref	
30–50	66	1.90 (±0.21)	0.22	0.23	0.13–0.31	0.001
50–70	87	2.11 (±0.22)	0.43	0.44	0.35–0.52	0.001
70 or more	39	2.20 (±0.26)	0.52	0.53	0.43–0.63	0.001
Gender						
Male	124	2.06 (±0.28)	Ref	Ref	Ref	
Female	101	1.93 (±0.26)	−0.12	−0.14	−0.19 (−0.08)	0.001

**Table 4:** **Mean difference of abdominal aorta diameters based on age, gender, and other factors at** L3 **vertebral level**

Characteristics	Number	Mean (SD)	Mean difference (crude) (coef.)	Mean difference (adjusted) (coef.)	95%CI	*P*-value
Age (years)						
Lower than 30	33	1.31 (±0.21)	Ref	Ref	Ref	
30–50	66	1.42 (±0.16)	0.10	0.11	0.02–0.18	0.008
50–70	87	1.55 (±0.19)	0.23	0.22	0.15–0.30	0.001
70 or more	39	1.59 (±0.26)	0.27	0.23	0.13–0.32	0.001
Gender						
Male	124	1.54 (±0.21)	Ref	Ref	Ref	
Female	101	1.41 (±0.20)	−0.12	−0.11	−0.17 (−0.07)	0.001
Alcohol consumption						
No	159	1.45 (±0.22)	Ref	Ref	Ref	
Yes	66	1.55 (±0.19)	0.09	0.07	0.01–0.12	0.010
Diabetic status of patients						
No	191	1.46 (±0.21)	Ref	Ref	Ref	
Yes	34	1.62 (±0.18)	0.16	0.08	0.00–0.16	0.032

A strong positive correlation was observed between age and mean abdominal aortic diameter at both the suprarenal (*r* = 0.98, *P* < 0.05) and infrarenal levels (*r* = 0.99, *P* < 0.05), suggesting that aortic diameter increases consistently with age. This age-related aortic dilation may have important clinical implications for determining appropriate intervention thresholds in screening programs.

These findings are in line with previous studies that also report a significant influence of age and gender on aortic size. However, the relatively smaller mean diameters observed in our study, especially among asymptomatic Asian individuals, further support the hypothesis that current intervention thresholds – largely based on Western populations – may require reassessment. This emphasizes the need for population-specific criteria, particularly in Asian cohorts, where smaller baseline aortic sizes could imply a lower threshold for aneurysmal risk.

## Discussion

The dilation of the AA is known as an AAA. When the AA’s diameter increases by more than 50%, ectasia develops, which then progresses to the development of an aneurysm condition[6]. However, an AAA is defined as an increase in diameter of more than 3 cm based on angiographic investigations[7]. As people age, the risk of aneurysm rupture rises[8]. Ruptures of AAAs usually result in severe hypotension, and while 50% of patients with ruptured AAAs make it to the hospital alive, up to 50% do not survive treatment^[9,10]^.

A variety of imaging modalities, including computed tomography (CT), angiography, magnetic resonance imaging, and ultrasonography, are available to screen or detect early aberrant changes in the size of the AA. These days, one of the most used noninvasive medical procedures that offers comprehensive information on the aorta and its branches is CT angiography. This is because the procedure is dependable and informative[11]. A CT scan can determine the size of an artery using a variety of techniques[12]. An examination using triple phase contrast is used to diagnose abdominal diseases, among which the TD of the AA is measured using the arterial phase. Using a CT scan, this study determined the typical diameters of the AA in the Nepalese population and examined how the aortic diameter varied with age, gender, and other health-related factors at two different vertebral levels.

The mean diameter of the suprarenal AA (T12 vertebral level) was 2.006 cm (±0.28), and the mean diameter of the infrarenal aorta (L3 vertebral level) was 1.486 cm (±0.22). These observations support the fact that the diameter of the AA decreases as it courses inferiorly^[1,13]^. The mean AA diameters showed a progressive increase with advancing age at both suprarenal and infrarenal levels. Similar findings have been reported by previous studies that measured aortic diameters while considering age^[14–16]^.

One of the major factors affecting the diameter of the AA is gender[1]. In our study, the AA diameter of males was larger than females at both suprarenal and infrarenal levels. This finding was consistent with previous studies that suggested that males have larger AA diameters than females^[2,14]^.

In our study, the mean diameter of the AA at the suprarenal or T12 vertebral level was 2.06 cm (±0.28) in male participants and 1.93 cm (±0.26) in female participants. The mean diameter of the AA at the infrarenal or L3 vertebral level was 1.54 cm (±0.21) in male participants and 1.41 cm (±0.20) in female participants. These findings are smaller compared to studies conducted in Australia, where the normal reference values for infrarenal aortic diameters were 16–18 mm for women and 19–21 mm for men[14]. Similarly, a study conducted in Turkey found that the mean subdiaphragmatic aortic diameter was 19 ± 4 mm in men and 18 ± 3 mm in women, while the mean aortic diameters at the level of bifurcation were 16 ± 4 mm in men and 15 ± 3 mm in women[17]. In a study conducted in the Netherlands with the elderly population with a mean age of 67 years, the mean infrarenal aortic diameter was 19.5 mm for men and 17 mm for women[18]. In comparison, our study had a mean age of 50.9 years and observed smaller abdominal aortic diameters. Notably, even among the elderly subgroup of our participants (aged >70 years), the mean infrarenal diameter was 1.59 cm (±0.26), which is still smaller than the measurements reported in the Netherlands.

In contrast, our results are more comparable to studies conducted in the Indian population, where the mean diameter of the suprarenal AA was 19.0 ± 2.3 mm in men and 17.1 ± 2.3 mm in women, and the mean diameter of infrarenal AA was 13.8 ± 1.9 mm in men and 12.0 ± 1.6 mm in women[14]. Similarly, a study conducted in the Pakistani population demonstrated that the mean AA diameter at the suprarenal level was 21.47 ± 2.25 mm in men and 19.18 ± 2.31 mm in women[19]. Another study also conducted in the Pakistani population found the mean transverse AA diameter at the infrarenal level was 16.51 ± 2.21 mm in men and 14.03 ± 1.64 mm in women[20].

The results demonstrate that Nepalese individuals have smaller abdominal aortic diameters than Western populations but are comparable to South Asian populations. Nepal’s distinctive lifestyle and geographic features, especially its high altitude and traditional diet of complex carbohydrates and minimal saturated fats, may be responsible for its unusual patterns of vascular remodeling. The Nepalese population’s baseline aortic diameters may be impacted by prolonged exposure to chronic hypoxia at higher elevations, which can also impair vascular tone and structural adaptability.

According to the European Society for Vascular Surgery guidelines, the intervention threshold ranges from 5.0 to 5.5 cm[21]. However, given the generally smaller aortic diameters in the Asian population, a lower threshold for intervention may be warranted. This study further supports the need for a reassessment of size-based criteria in Asian cohorts. In a meta-analysis of normal infrarenal aortic diameter in the general world population, the pooled mean AA diameter was 20.1 mm in men and 17.8 mm in women, with the largest mean diameter observed in Oceania (22.9 mm) and the smallest in Asia (18.4 mm)[22]. These differences highlight the importance of establishing population-specific reference values for the accurate diagnosis and management of aortic diseases. These anatomical differences underscore the need for region-specific clinical approaches to vascular surgery and device selection. Based on the average infra-renal aortic diameter of 1.54 cm at the L3 vertebral level in Nepalese males, it is recommended that aortic graft sizes in the range of 16–20 mm be maintained in Nepal. This recommendation aligns more closely with the anatomical characteristics of the local population and may help prevent complications associated with the use of oversized grafts designed for Western populations.

Recent advancements in artificial intelligence (AI) have transformed the landscape of medical research and diagnostics, moving beyond descriptive analytics toward predictive and personalized intervention. AI models trained on medical imaging, such as AlphaFold, have already revolutionized molecular biology and drug discovery by accurately predicting protein structures[23]. Similarly, AI algorithms using clinical imaging are being developed to provide tailored diagnostic and therapeutic strategies for oncology patients AI model using clinical images for genomic prediction and tailored treatment in patients with cancer[24].

Our study provides important race-specific data on abdominal aortic size in the Nepalese population, which can help train AI models for more accurate vascular diagnosis. Most current AI systems use Western data, which may not apply well to Asian populations and could lead to misdiagnosis. Our findings can help adjust aneurysm detection thresholds and guide personalized surgical planning. The links we found between aortic size and factors like age, diabetes, and alcohol use also give AI models useful inputs for predicting future risk. This moves us closer to using AI for personalized, real-time care in vascular medicine.

### Strengths and limitations

This cross-sectional study established the normal range of AA diameter in the Nepalese population and evaluated their relationship with demographic and health-related factors. However, the single-center design of the study is a limitation. Future research should be conducted with a multi-center approach and a larger population with diversity in terms of age and ethnicity.

## Conclusion

We established normal reference values for AA diameters in the Nepalese population. The mean diameter of the suprarenal AA measured at the T12 vertebral level was 2.06 cm (±0.28) in men and 1.93 cm (±0.26) in women. The mean diameter of the infrarenal aorta measured at the L3 level was 1.54 cm (±0.21) in men and 1.41 cm (±0.20) in women. We recommend adopting aortic graft sizes of 16–20 mm in Nepalese males to better match population-specific anatomy and avoid reliance on oversized imported grafts. The study concludes that the mean abdominal aortic diameter of the Nepalese population seems to be smaller than the Western population but comparable to the South Asian population. Age and gender of participants showed a statistically significant association with the diameter of the suprarenal AA. Additionally, age, gender, alcohol consumption, and diabetic status were statistically significantly associated with the diameter of the infrarenal AA. We recommend further studies with larger sample sizes to gain a deeper understanding of these findings and advance the diagnosis and management of aortic diseases.

## Data Availability

Data sharing is not applicable to this article.

## Research registration unique identifying number (UIN)

This study has been registered in the Research Registry.
